# Association between relative muscle strength and hypertension in middle-aged and older Chinese adults

**DOI:** 10.1186/s12889-023-17007-6

**Published:** 2023-10-25

**Authors:** Jin-hua Luo, Tu-ming Zhang, Lin-lin Yang, Yu-ying Cai, Yu Yang

**Affiliations:** 1 Geriatrics Research Unit, Affiliated Hospital of Guangdong Medical University, Zhanjiang, 524000 China; 2 Department of Geriatrics, Affiliated Hospital of Guangdong Medical University, Zhanjiang, 524000 China

**Keywords:** Muscle quality, Hypertension, Middle-aged and older adults, Relative muscle strength

## Abstract

**Background:**

The association between muscle defects and hypertension is well-established. However, the absence of pertinent and uncomplicated clinical indicators presents a challenge. Relative muscle strength (RMS) may offer a viable indicator. This study aimed to explore the association between RMS and hypertension.

**Methods:**

A total of 12,720 individuals aged ≥ 45 years from the 2011 wave of the China Health and Retirement Longitudinal Study (CHARLS) were included. Grip strength was recorded and appendicular skeletal muscle mass (ASM) was estimated using a validated mathematical formula. The RMS was calculated as the ratio of grip strength to ASM. Hypertension was determined based on previous diagnosis, history of hypertension medication use, and current blood pressure. Logistic regression models were employed to investigate the relationship between RMS and hypertension.

**Results:**

The prevalence of hypertension was 41.7% (5,307/12,720 patients). RMS was negatively correlated with hypertension with an OR (95% CI) of 0.68 (0.59–0.79) for males, 0.81 (0.73–0.90) for females, and 0.78 (0.72–0.85) for the entire population after adjusting for related covariates including age, education, marital history, smoking history, drinking history, diabetes, hyperlipidemia, and obesity. The trend test showed a linear association among males, females, or the entire population. Stratified analysis showed a consistent negative correlation between RMS and hypertension.

**Conclusions:**

Higher RMS is an independent protective factor against hypertension and efforts to promote RMS may be beneficial for the prevention and management of hypertension.

**Supplementary Information:**

The online version contains supplementary material available at 10.1186/s12889-023-17007-6.

## Background

Hypertension is a risk factor for cardiovascular diseases [1]. Worldwide, the prevalence of hypertension among those reporting a previous diagnosis in 2019 was 59% (55–62) among females and 49% (46–52) among males, with only 47% (43–51) of females and 38% (35–41) of males being treated [2]. Prevention and management of hypertension can reduce the risk of myocardial infarction and stroke, as well as related disability and mortality, thus greatly reducing the economic burden on health care [3]. Despite a better understanding of hypertension and improvements in therapeutic strategies, the incidence of hypertension-related complications of cardiovascular diseases continues to increase [4]. Thus, the risk factors for hypertension need to be explored further.

Muscle quality, as distinguished from muscle quantity or mass, is gaining recognition as a means of elucidating and characterizing intricate intramuscular alterations linked to muscle functionality during the aging process [5]. Evidence increasingly suggests that a decline in muscle quality increases the risk of hypertension. Sarcopenia is an age-related clinical condition caused by the loss of muscle quality. A meta-analysis showed an increased risk of hypertension in subjects with sarcopenia compared with those without (OR = 1.39, 95% CI: 1.15–1.67, *p* < 0.01) [6]. Myosteatosis, or ectopic adipose infiltration in the skeletal muscle, is one of the manifestations of decreased muscle tissue quality. This increases the degree of local and systemic insulin resistance [7], which in turn, may contribute to hypertension [8]. Advanced myosteatosis measured using abdominal computed tomography (CT) at the L3 level was significantly associated with a higher risk of hypertension even after adjusting for relevant covariates [9]. It should be noted that muscle quality is recognized as a modifiable risk factor, as appropriate strength exercise can improve muscle quality in various populations [10, 11]. Therefore, it is sensible to begin with muscle quality to develop preventive strategies for health problems such as hypertension. However, the diagnostic process of sarcopenia is complex, limiting its clinical applicability. Moreover, the clinician-friendliness of identifying myosteatosis using equipment such as CT and other specific techniques is questionable. Therefore, there is an urgent need to identify clinically accessible indicators of muscle quality.

Relative muscle strength (RMS), defined as muscle power per unit of muscle mass, is comparable to myosteatosis as a metric of muscle quality [5, 12, 13]. RMS is recommended for the characterization of muscle quality in clinical studies. In most studies, upper limb muscle quality is typically calculated as the ratio of grip strength to lean mass in the arms [14, 15]. Lower limb muscle quality is calculated as the ratio of quadriceps strength to lean mass in the legs [15]. Alternatively, muscle quality can be determined through grip strength in relation to appendicular skeletal muscle mass (ASM), that is, the lean mass in both arms and legs [16]. Among the calculated parameters associated with RMS, grip strength is a frequently used indicator of muscle strength in healthcare settings, and ASM is routinely used to diagnose sarcopenia, making grip strength/ASM a more readily accessible measure for clinical use. Several studies have reported an association between RMS and health problems; Park et al. found that RMS was significantly lower in older adults with diabetes than in those without diabetes [17]. Yoon et al. found that the worse the glucose metabolism, the lower the RMS was [18]. In addition, the higher the RMS, the lower the risk of cognitive impairment in men [16]. Among patients undergoing dialysis, a lower RMS is an independent predictor of increased mortality [19]. However, there is currently no research indicating a connection between RMS and hypertension. Therefore, this study aimed to investigate the correlation between RMS and the risk of hypertension using data from the China Health and Retirement Longitudinal Study (CHARLS).

## Methods

### Study design and participants

**Fig. 1:**
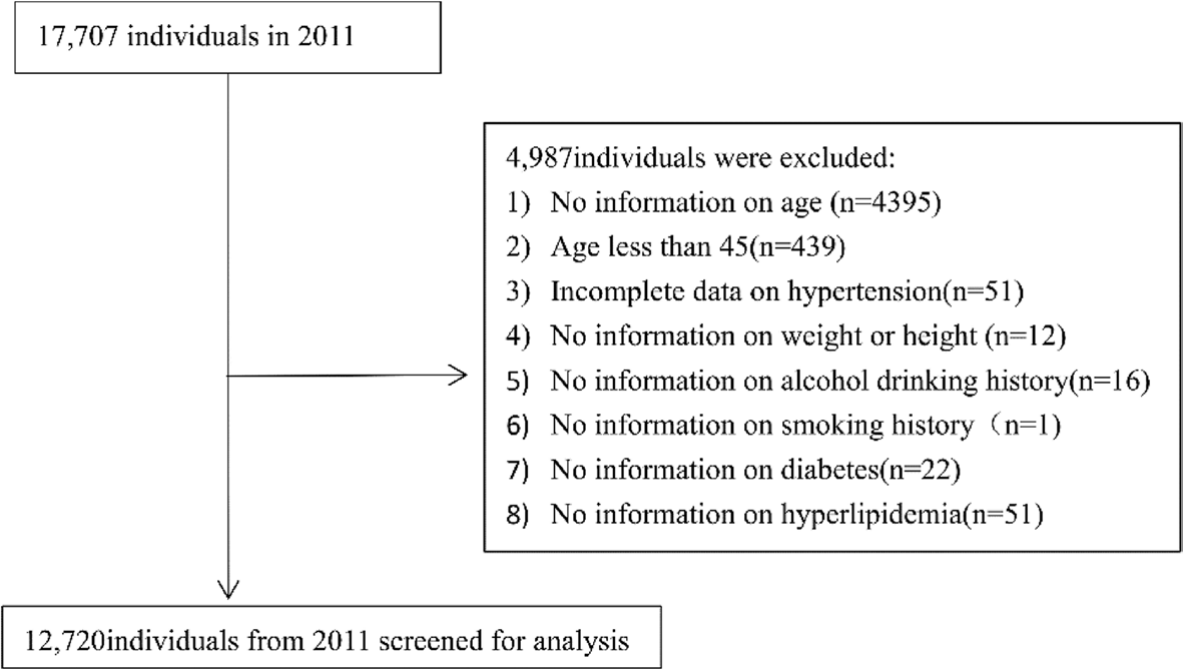
Flow chart of the study

The aim of the CHARLS was to investigate the current state of aging throughout China and facilitate multidisciplinary research on aging using high-quality microdata collected from Chinese households and individuals over 45 years of age. A multistage proportional to size sampling method was used for the baseline survey of the CHARLS in 2011, and three follow-up studies were conducted in 2013, 2015, and 2018 [20, 21, 22]. The CHARLS dataset is available at http://charls.pku.edu.cn/en. The study was approved by the Peking University Biomedical Ethics Committee and informed consent forms were signed by all participants. The data for this study were retrieved from a baseline survey conducted in 2011, which included 17,707 participants. The inclusion criteria were as follows: 1) age ≥ 45 years and 2) complete blood pressure measurement data. Exclusion criteria were as follows: 1) no information on weight or height, 2) no information on drinking or smoking history, and 3) no information on a diagnosis of diabetes or hyperlipidemia (Fig. 1).

### Diagnosis of hypertension

Hypertension was diagnosed based on the following conditions: (1) systolic blood pressure ≥ 140 mmHg, (2) diastolic blood pressure ≥ 90 mmHg, (3) self-reported history of hypertension, and (4) self-reported intake of anti-hypertensive drugs [20, 23].

### Assessment of RMS

RMS was defined as handgrip strength/ASM. To measure the grip strength (kg) of the dominant and non-dominant hands, the participants were asked to squeeze the dynamometer (TMWL-1000; Yuejian, China) as hard as possible. The maximum value was recorded after testing both hands twice, using a dynamometer at a right angle. A previously reported mathematical formula was used to estimate ASM in the Chinese population [24–27]: ASM = 0.193 × weight (kg) + 0.107 × height (cm) – 4.157 × sex – 0.037 × age (year) – 2.631. Sex was set to one if male; otherwise, it was set to two.

### Potential covariates

Sociodemographic characteristics included age, sex, education (junior high school and below, high school/vocational high school, college and above), and marital status (married/other, including separated, unmarried, divorced, and widowed). Health-related factors included smoking history (no for nonsmoker/yes for former smoker or current smoker), drinking history (no for nondrinker/yes for former drinker or current drinker), diabetes (self-reported diabetes diagnosis history or use of hypoglycemic drugs or fasting glucose ≥ 7 mmol/L or glycosylated hemoglobin ≥ 6.5%), hyperlipidemia (self-reported hyperlipidemia diagnosis history or use of lipid-lowering drugs or blood total cholesterol ≥ 240 mg/dL or low density cholesterol ≥ 160 mg/dL or triglyceride ≥ 200 mg/dL or high density cholesterol < 40 mg/dL), and obesity (BMI ≥ 28 kg/m^2^).

### Statistical analysis

All analyses were performed using the R statistical software package (http://www.R-project.org) and Free Statistics software version 1.8 (http://www.clinicalscientists.cn/freestatistics). Quantitative data with normal distribution were expressed as mean ± standard deviation and median (interquartile, IQR) in cases of skewed distribution. The comparison of groups was conducted using analysis of variance and the Kruskal–Wallis rank-sum test. The qualitative data were summarized in terms of percentages and subsequently compared utilizing the chi-square test. Additionally, logistic regression analysis was conducted to evaluate the association between RMS and hypertension. Regression analyses included unadjusted and adjusted models (adjusted for covariates, including age, education, marital status, smoking history, drinking history, diabetes, hyperlipidemia, and obesity). *P* < 0.05 was considered statistically significant.

## Results

### Characteristics of participants

A total of 12,720 individuals were finally included in the analysis: 6081 (47.8%) male and 6639 (52.2%) female. The prevalence of hypertension was 41.7% (5,307/12,720 individuals). The mean RMS was 1.9, with a standard deviation of 0.5. Table 1 shows the characteristics of the participants according to hypertension status. Individuals with hypertension were more likely to be older, obese, and had hyperlipidemia and diabetes, whereas individuals without hypertension had higher grip strength, lower BMI, lower ASM, and higher RMS.

**Table 1:** Characteristic of participants

Variables	Total (*n* = 12,720)	Without hypertension (*n* = 7413)	With hypertension (*n* = 5307)	*p*
Age, median (IQR), years	58.0 (52.0, 65.0)	56.0 (50.0, 63.0)	61.0 (55.0, 69.0)	< 0.001
Sex, *n* (%)				0.002
Male	6081 (47.8)	3629 (49)	2452 (46.2)	
Female	6639 (52.2)	3784 (51)	2855 (53.8)	
Education, *n* (%)				< 0.001
Below lower secondary	11,397 (89.6)	6591 (88.9)	4806 (90.6)	
Upper secondary & vocational training	1144 (9.0)	725 (9.8)	419 (7.9)	
Tertiary	179 (1.4)	97 (1.3)	82 (1.5)	
Marital status, *n* (%)				< 0.001
Married	10,490 (82.5)	6316 (85.2)	4174 (78.7)	
Others	2230 (17.5)	1097 (14.8)	1133 (21.3)	
Drinking, *n* (%)				0.837
No	7467 (58.7)	4346 (58.6)	3121 (58.8)	
Yes	5253 (41.3)	3067 (41.4)	2186 (41.2)	
Smoking, *n* (%)				0.248
No	7604 (59.8)	4400 (59.4)	3204 (60.4)	
Yes	5116 (40.2)	3013 (40.6)	2103 (39.6)	
BMI, mean ± SD	23.5 ± 4.0	22.8 ± 3.6	24.4 ± 4.3	< 0.001
Obesity, *n* (%)				< 0.001
No	11,298 (88.8)	6908 (93.2)	4390 (82.7)	
Yes	1422 (11.2)	505 (6.8)	917 (17.3)	
Hyperlipidemia, *n* (%)				< 0.001
No	8360 (65.7)	5270 (71.1)	3090 (58.2)	
Yes	4360 (34.3)	2143 (28.9)	2217 (41.8)	
Diabetes, *n* (%)				< 0.001
No	10,902 (85.7)	6642 (89.6)	4260 (80.3)	
Yes	1818 (14.3)	771 (10.4)	1047 (19.7)	
Grip strength, mean ± SD	32.5 ± 10.4	33.2 ± 10.2	31.5 ± 10.7	< 0.001
ASM, mean ± SD	17.3 ± 4.2	17.2 ± 4.0	17.5 ± 4.4	< 0.001
RMS, mean ± SD	1.9 ± 0.5	1.9 ± 0.5	1.8 ± 0.5	< 0.001

*IQR* Interquartile range, *SD* Standard deviation, *BMI* Body mass index, *ASM* Appendicular skeletal muscle mass, *RMS* Relative muscle strength

### Association between RMS and hypertension

**Fig. 2:**
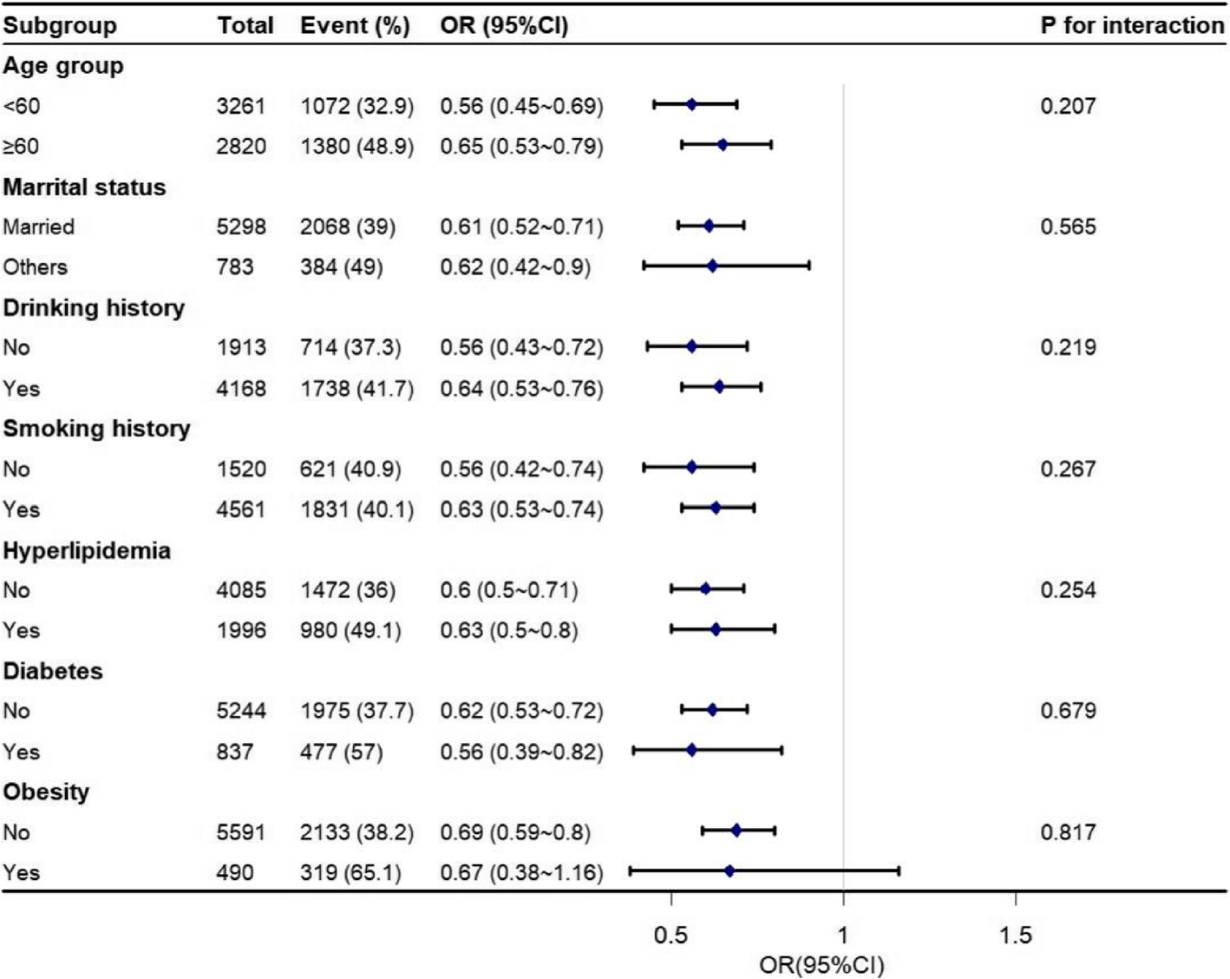
Forest plot for the association between RMS and hypertension in different subgroup of male population. Odd ratios (ORs) were adjusted for age, education, marital status, smoking history, drinking history, diabetes, hyperlipidemia, obesity

**Fig. 3:**
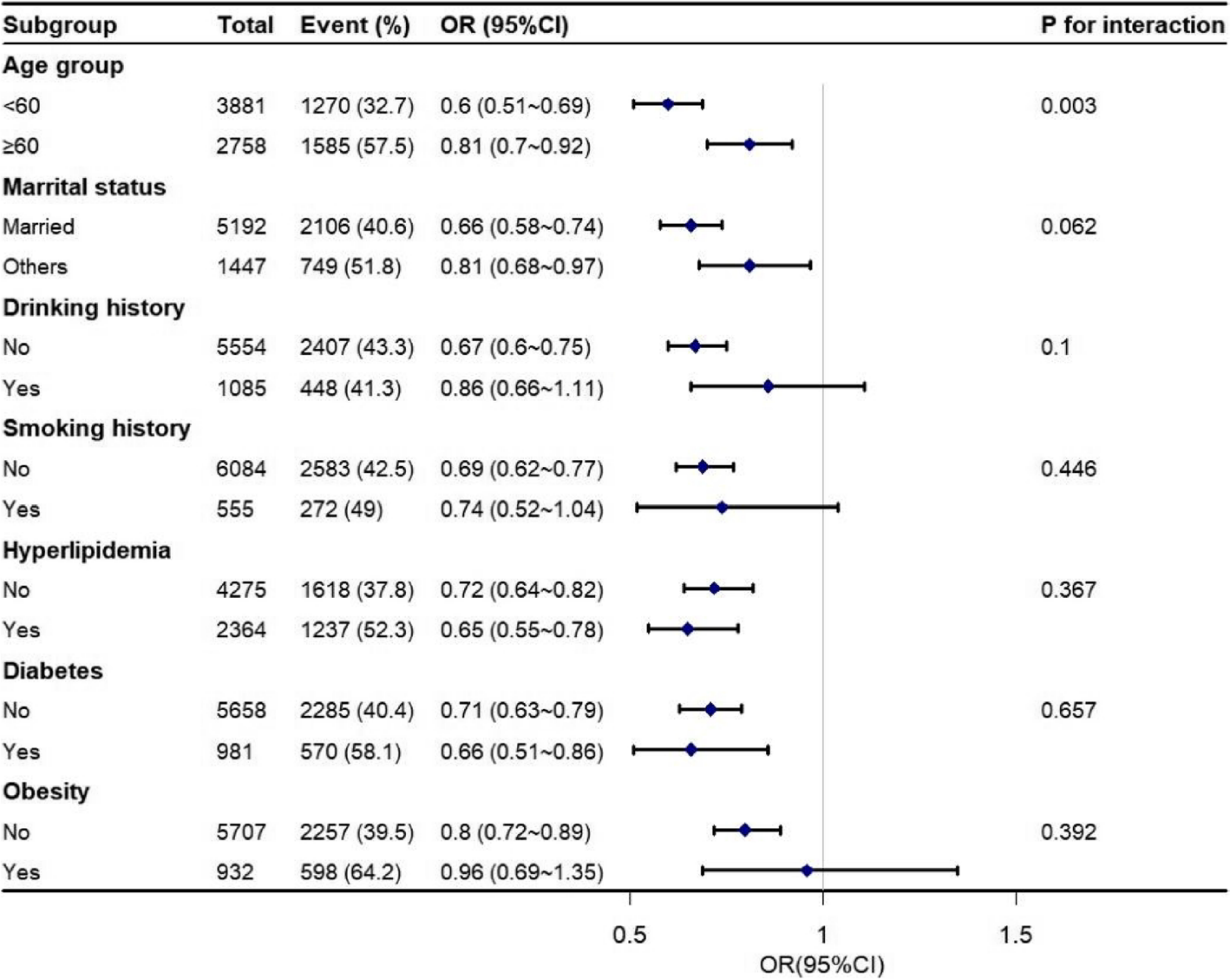
Forest plot for the association between RMS and hypertension in different subgroup of female population. Odd ratios (ORs) were adjusted for age, education, marital status, smoking history, drinking history, diabetes, hyperlipidemia, obesity

Logistic regression analysis showed that RMS was associated with hypertension with an OR (95% CI) of 0.45 (0.39–0.51) in males, 0.6 (0.54–0.66) in females and 0.54 (0.50–0.58) in the entire population in the unadjusted model. After adjusting for confounders, RMS remained associated with hypertension negatively, with an OR (95% CI) of 0.68 (0.59–0.79) in males, 0.81(0.73–0.90) in females, and 0.78(0.72–0.85) in the entire population (Table 2). The linear association between RMS and hypertension was further explored using a trend test in which participants were grouped according to tertiles of RMS. Results showed that the prevalence of hypertension was significantly lower in those with the highest RMS tertile than in those with the lowest RMS tertile in fully adjusted models among males (OR: 0.74, 95% CI: 0.64–0.84, *P* for trend < 0.001), females (OR: 0.75, 95% CI: 0.66–0.85, *P* for trend < 0.001), and the entire population (OR: 0.76, 95% CI: 0.70–0.84, *P* for trend < 0.001). In addition, subgroup analysis revealed a consistent negative correlation between RMS and hypertension among individuals of different sexes when stratified by age, marital status, smoking history, drinking history, diabetes, hyperlipidemia, and obesity status, with ORs below the reference value. Furthermore, a significant interaction (*P* for interaction: 0.003) was observed when the analyses of hypertension were stratified by age among females, such that the risk of hypertension by RMS was higher among those in the middle-aged group than among those in the older group (Figs. 2 and 3).

**Table 2:** Association between RMS and hypertension in middle-aged and older adults

Variables	Male (*n* = 6081)		Female (*n* = 6639)		All (*n* = 12,720)	
	**OR (95% *CI*)**	***P***	**OR (95% *CI*)**	***P***	**OR (95% *CI*)**	***P***
**RMS**^**a**^	0.45 (0.39–0.51)	< 0.001	0.60 (0.54–0.66)	< 0.001	0.54 (0.50–0.58)	< 0.001
**RMS**^**b**^	0.68 (0.59–0.79)	< 0.001	0.81 (0.73–0.90)	< 0.001	0.78 (0.72–0.85)	< 0.001
**RMS tertiles**^**b**^						
**1st**	1.00 (Ref)		1.00 (Ref)		1.00 (Ref)	
**2nd**	0.83 (0.73–0.95)	0.006	0.80 (0.70–0.91)	0.001	0.83 (0.75–0.91)	< 0.001
**3rd**	0.74 (0.64–0.84)	< 0.001	0.75 (0.66–0.85)	< 0.001	0.76 (0.70–0.84)	< 0.001
***P* for trend**		< 0.001		< 0.001		< 0.001

^a^unadjusted model

^b^model adjusted for age, education, marital status, smoking history, drinking history, diabetes, hyperlipidemia, and obesity (plus sex for the entire population)

## Discussion

Our findings show that RMS is negatively and linearly correlated with hypertension in both males and females and in the entire population, even after adjusting for covariates. In other words, a higher RMS value reduced the risk of developing hypertension. Stratified analysis further showed that the correlation between RMS and hypertension was robust across subgroups with different ages, marital history, smoking history, diabetes history, hyperlipidemia history, and obesity status.

To the best of our knowledge, few studies have examined the association between RMS and hypertension. However, the relationship between the parameters used to calculate RMS, that is, grip strength, muscle mass, and hypertension, has been reported in several studies. Studies have shown that higher grip strength is independently associated with lower blood pressure and is therefore a protective factor against hypertension [28, 29]. In contrast, another study showed that higher grip strength was associated with higher blood pressure after adjusting for BMI [30]. However, we did not observe any correlation between grip strength and hypertension (Table S1). These inconsistent results may be attributed to significant differences in age and BMI distribution among the populations in these studies. Similarly, there is no consensus regarding the association between muscle mass and hypertension. Julius et al. were the first to report a positive correlation between skeletal muscle mass of the extremities and blood pressure [31]. Vaziri et al. also found a positive correlation between skeletal muscle mass and blood pressure independent of BMI in a younger population [32]. Liu et al. found that the association between muscle mass and incident hypertension followed a J-shaped curve in a community-dwelling population of older Chinese adults [33]. A study by Ye et al. also showed that muscle mass, or height- and weight-corrected muscle mass, were positively associated with blood pressure and hypertension, suggesting that elevated muscle mass may be a risk factor for hypertension [34]. However, Han et al. reported that low muscle mass was correlated with a higher risk of developing hypertension among Korean men, whereas this correlation was not significant in women [35]. We found in this study that muscle mass (ASM) and BMI-corrected muscle mass (ASMI) were positively associated with hypertension in both men and women (Table S1). These discrepancies could be the result of obesity, as obese individuals have higher muscle mass. RMS, which combines grip strength and muscle mass, showed a stable and independent correlation with hypertension. This is likely due to the fact that RMS reflects changes in muscle properties and is not susceptible to factors affecting muscle mass and grip strength, such as BMI, height, and weight. However, it should be noted that while the correlation between RMS and hypertension was maintained among the males after replacing obesity status in the model with BMI, this association disappeared in the female population after making the above substitution (Table S2). This suggests that a higher level of obesity in women than in men has a greater impact on the development of hypertension, thus attenuating the association between RMS and hypertension. Furthermore, within the subgroup analysis focusing on female individuals, the correlation between RMS and hypertension was stronger in the middle-aged cohort (aged 45–59 years). Since the average age of menopause among Chinese women is reported to be 49.3 ± 3.3 years, with a median age of 50 years [36], one possible explanation for this phenomenon could be the fact that some middle-aged women may not have reached menopause, resulting in comparatively elevated levels of estrogen [37]. Estrogen has been shown to offer protection against hypertension [38].

However, the mechanism underlying the association between RMS and hypertension remains unclear. RMS is closely related to myosteatosis [39], which affects muscle strength and reduces RMS [40, 41]. This leads to the accumulation of diacylglycerol and ceramides in muscle cells, thereby inhibiting glucose uptake by the muscles and gluconeogenesis and exacerbating insulin resistance [42–44]. Given that insulin resistance is closely associated with the risk of developing hypertension [45–47], the correlation between RMS and hypertension may be related to myosteatosis-induced insulin resistance. Previous studies have shown that RMS is closely associated with muscle insulin resistance [48]. Consistent with this, we found that a low RMS was associated with a worse glucolipid metabolic profile (Table S3), which is a parameter associated with insulin resistance. Hyperinsulinemia due to insulin resistance leads to sympathetic excitation, decreased renal vasoconstriction, and increased renal tubular sodium reabsorption, causing an increase in blood pressure [49, 50]. Furthermore, low muscle quality and hypertension share common risk factors such as older age, sedentary lifestyle [51, 52], and obesity [53, 54]. Nevertheless, the cross-sectional results did not elucidate a causal association, which may be an effect of confounding factors and requires further validation.

This study has several strengths. First, the inclusion of a substantial proportion of the population ensures national representation. Second, multiple research groups validated the quality of the data used in this study. Finally, this study represents an initial exploration of the correlation between RMS and hypertension. However, it is important to acknowledge the limitations of this study. First, owing to its cross-sectional nature, the causal relationship between RMS and hypertension could not be confirmed, and requires further investigation through longitudinal studies. Second, despite adjusting for numerous common confounders, certain factors such as genetic predisposition, sodium intake, and physical activity were not considered. Lastly, the study focused solely on a Chinese population; thus, caution must be exercised when generalizing the findings to other populations, particularly given the apparent population differences in muscle quality [55].

## Conclusions

Higher RMS is an independent protective factor against hypertension, and measures to increase RMS in middle-aged and older adults may be beneficial for the prevention and management of hypertension. It is notable that RMS may serve as a more accessible and consistent metric than other muscle parameters when assessing the relationship between muscle impairment and hypertension.

### Supplementary Information

**Additional file 1:** **Table S1.** Association between ASM, ASMI, grip strength and hypertension in middle-aged and older adults. **Table S2.** Association between RMS and hypertension in middle-aged and older adults. **Table S3.** Prevelance of diabetes and hyperlipidemia in different RMS groups.

## Data Availability

The data that support this study are available from the websites of CHARLS (http://charls.pku.edu.cn).

## Abbreviations

CHARLS: China Health and Retirement Longitudinal Study
RMS: Relative muscle strength
ASM: Appendicular skeletal muscle mass
ASMI: Appendicular skeletal muscle mass index
BMI: Body mass index
OR: Odds ratio
IQR: Interquartile
SD: Standard deviation
