# Supplementary material for: Association between relative muscle strength and hypertension in middle-aged and older Chinese adults
Source: BMC Public Health. 2023 Oct 25;23:2087. doi: 10.1186/s12889-023-17007-6 (PMC10598916; doi:10.1186/s12889-023-17007-6)
Supplement: Supplementary file 1 — Additional file 1: Table S1. Association between ASM, ASMI, grip strength and hypertension in middle-aged and older adults. Table S2. Association between RMS and hypertension in middle-aged and older adults. Table S3. Prevelance of diabetes and hyperlipidemia in different RMS groups. [file 12889_2023_17007_MOESM1_ESM.docx]

**Table S1** Association between ASM, ASMI, grip strength and hypertension in middle-aged and older adults

| **Variable** | **n.total** | **n.event_%** | **Model 1** | | **Model 2** | |
| --- | --- | --- | --- | --- | --- | --- |
|  |  |  | **OR（95%*CI*）** | ***p*** | **OR（95%*CI*）** | ***p*** |
| **Male** |  |  |  |  |  |  |
| ASM | 6081 | 2452 (40.3) | 1.10 (1.08–1.12) | <0.001 | 1.17 (1.14–1.20) | <0.001 |
| Grip strength | 6081 | 2452 (40.3) | 0.99 (0.98–0.99) | <0.001 | 1.00 (1.00–1.01) | 0.164 |
| **Female** |  |  |  |  |  |  |
| ASM | 6639 | 2855 (43.0) | 1.04 (1.03–1.06) | <0.001 | 1.13 (1.10–1.16) | <0.001 |
| Grip strength | 6639 | 2855 (43.0) | 0.97 (0.97–0.98) | <0.001 | 1.00 (0.99–1.01) | 0.521 |
| ALL* |  |  |  |  |  |  |
| ASM | 12720 | 5307 (41.7) | 1.02 (1.01–1.03) | <0.001 | 1.15 (1.13–1.17) | <0.001 |
| Grip strength | 12720 | 5307 (41.7) | 0.984 (0.981–0.988) | <0.001 | 1.005 (0.99–1.01) | 0.055 |

Model 1: not adjusted; Model 2: adjusted for: age, education, marital status, drinking, smoking, Hyperlipidemia, diabetes, obesity( *: plus sex).

**Table S2** Association between RMS and hypertension in middle-aged and older adults

| **Group** | **Model 1** | | **Model 2** | |
| --- | --- | --- | --- | --- |
|  | **OR（95%*CI*）** | ***P*** | **OR（95%*CI*）** | ***P*** |
| **Male** | 0.45 (0.39–0.51) | <0.001 | 0.79 (0.68–0.92) | 0.003 |
| **Female** | 0.6 (0.54–0.66) | <0.001 | 0.94 (0.85–1.04) | 0.233 |

Model 1: not adjusted; Model 2: adjusted djusted for: age, education, marital status, drinking, smoking, Hyperlipidemia, diabetes, BMI.

Table S3 Prevelance of diabetes and hyperlipidemia in different RMS groups.

| **Variables** | **Total (n = 12720)** | **Q1 (n =3180)** | **Q2 (n = 3179)** | **Q3 (n = 3180)** | **Q4 (n = 3181)** | ***p*** |
| --- | --- | --- | --- | --- | --- | --- |
| **Male** | 6081 |  |  |  |  |  |
| Diabetes, n (%) | 837 (13.8) | 276 (18.2) | 219 (14.4) | 188 (12.4) | 154 (10.1) | < 0.001 |
| Hyperlipidemia, n (%) | 1996 (32.8) | 558 (36.7) | 523 (34.4) | 469 (30.9) | 446 (29.3) | < 0.001 |
| **Female** | 6639 |  |  |  |  |  |
| Diabetes, n (%) | 981 (14.8) | 344 (20.7) | 254 (15.3) | 210 (12.7) | 173 (10.4) | < 0.001 |
| Hyperlipidemia, n (%) | 2364 (35.6) | 681 (41) | 620 (37.4) | 572 (34.5) | 491 (29.6) | < 0.001 |

Q1, Q2, Q3, Q4: Groups of RMS by quartiles
